# Society Meetings

**Published:** 1898-02

**Authors:** 


### Society Meetings.

The Medical Society of the State of New York held its ninety-second annual meeting Tuesday, Wednesday and Thursday, January 25, 26 and 27, 1898, in the city hall, Albany, commencing at 9.15 A. M. on the 25th and ending at 1 P. M. on the 27th, under the presidency of Dr. Seneca D. Powell, of New York. The following program was observed :

TUESDAY MORNING SESSION, 9.15 O'CLOCK.

President's inaugural address, reports of officers and committees, executive business.

*Papers.*—Throat and nose affections and their relations to general medicine, Walter F. Chappell, New York ; Ear manifestations in general disease, Wendell C. Phillips, New York ; Cases of acute nondiphtheritic inflammation of the larynx, requiring the prolonged retention of the intubation tube, John O. Roe, Rochester ; The report of a case of unusual contraction of the visual field and disorder of the color sense following an injury, T. F. C. Van Allen, Albany ; The railway surgeon and his work, C. B. Herrick, Troy ; What shall the state and county do for the consumptive? John H. Pryor, Buffalo ; The advantages of state control in medicine, with results observed, William Warren Potter, Buffalo.

TUESDAY AFTERNOON SESSION, 2.15 O'CLOCK.

*Papers.*—A contribution to the study of melancholia, with a report of the examination of the blood in fifty-seven cases, B. C. Loveland, Clifton Springs ; The cold water treatment of typhoid fever in private practice, John T. Wheeler, Chatham ; Anemia, R. C. M. Page, New York ; On the vagaries and wanderings of gall-stones, with clinical reports, Henry L. Elsner, Syracuse ; The treatment of delirium, Joseph Collins, New York ; Paralysis — prognosis and treatment, Edward D. Fisher, New York ; The relation of bacteria to the normal alimentary canal, Herbert U. Williams, Buffalo ; The rivals of the physician in practice, Reynold W. Wilcox, New York ; The hygienic management of dairies, E. F. Brush, Mt. Vernon ; The municipal control of milk supply in cities and villages, with report of health regulation, T. B. Carpenter, Buffalo ; The present status of expert medical testimony, Evarts M. Morrell, Yonkers ; Expert testimony, J. B. Ransom, Dannemora.

TUESDAY EVENING SESSION, 7.15 O'CLOCK.

Practical exposition of the x-ray in medicine and surgery. Exhibition in charge of Arthur L. Fisk, of New York.

*Papers.*—Technique and apparatus, Samuel Lloyd, New York ; The x-ray in medicine, Dr. Williams, Boston ; The x-ray in surgery,

Arthur L. Fisk, New York; The x-ray, clinical experience, Wm. Hailes, Jr., Albany.

WEDNESDAY MORNING SESSION, 9.15 O'CLOCK.

*Papers.*—Some points in the technique of the Alexander operation, Herman E. Hayd, Buffalo; The present status of vaginal operations for diseases of the pelvic organs, Edwin B. Cragin, New York; Remote consequences of excessive uterine hemorrhage, W. E. Ford, Utica; Gauze drainage in laparatomy, Henry C. Coe, New York; The anatomy and function of the female perineum and the operation for its repair when lacerated, J. Riddle Goffe, New York; Traumatic injuries of the brain, J. H. Glass, Utica; Formaldehyde disinfection, E. H. Wilson, Brooklyn, discussed by Alvah H. Doty and Herman Biggs; The past, present and prospective methods of treatment of insanity in the state of New York, P. M. Wise, Albany.

WEDNESDAY AFTERNOON SESSION, 2.15 O'CLOCK.

*Papers.*—A year's work in appendicitis, Herman Mynter, Buffalo; The management of undescended testicle in hernia operation, William B. DeGarmo, New York; Investigation and exploration of the other kidney in contemplated nephrectomy, Geo. M. Edebohls, New York; Congenital dislocation of the shoulder backwards, with a report of seven cases and an operation for its relief, A. M. Phelps, New York; Discussion: The management of hypertrophy of the prostate gland and its complications. I. General consideration and catheter life, L. Bolton Bangs, New York; II. Prostatectomy and prostatotomy, supra-pubic and perineal, Samuel Alexander, New York; III. Bottini's galvano-caustic radical treatment, and the palliative treatment for hypertrophy of the prostate, Willy Meyer, New York; IV. Castration for the relief of hypertrophied prostate, L. S. Pilcher, New York; V. Stone, associated with hypertrophy of the prostate, E. L. Keyes, New York; Excision of the fibula for sarcoma, Samuel Lloyd, New York; The treatment of the fracture of the femur in children, Theodore Dunham, New York.

WEDNESDAY EVENING SESSION.

In the senate chamber at 7.45 o'clock. Anniversary address by the president. Reception by the president to the members, delegates and invited guests at the Albany Club at 9.30 o'clock.

THURSDAY MORNING SESSION, 9.30 O'CLOCK.

*Business Session and Reports of Committees.*

*Papers.*—Treatment of deficient excretion from kidneys not organically diseased, and some of the diseases peculiar to women, and diseases of the skin, L. Duncan Bulkley, New York; The relations of the physician to the practice of midwifery, C. F. Timmerman, Amsterdam; The conservative surgery of the fibroid tumor, A. H. Goelet, New York; Report of a case of osteotomy of both tibiæ and fibulæ for symmetrical antero-posterior angular deformity, F. H. Peck, Utica; Intra-tracheal injections for diseases of the bronchial tubes and lungs, H. S. Drayton, New York.

---

The Buffalo Academy of Medicine held meetings during the month of January, 1898, as follows:

*Section on Medicine.*—Tuesday evening, January 4th, program: Pulmonary disturbance in children; infantile asthma, by Dr. Irving M. Snow. Diagnosis of empyema, by Dr. Eugene A. Smith.

*Section on Surgery.*—Tuesday evening, January 11th, program: The parasitic origin of cancer, with demonstrations of the organisms and microscopical sections, by Dr. Roswell Park. Discussed by Drs. Herbert U. Williams and Woods Hutchinson.

*Section on Pathology.*—Tuesday evening, January 18th, program: The pathology of the cerebellum, by Dr. William C. Krauss. Exhibition of specimens, by Drs. Marcel Hartwig, A. L. Benedict and others. Exhibit of veterinary specimens and anomalies, by Dr. John T. Claris, V. S., inspector state board of health.

*Section on Obstetrics and Gynecology.*—Tuesday evening, January 25th, program: Subject to be announced, by Dr. James F. W. Ross, Toronto, Canada.

---

The Elmira Academy of Medicine held its annual meeting January 12, 1898. The retiring president, Dr. Frank W. Ross, chose for the subject of the annual address, Remarks on the use of the x-rays for diagnostic purposes with demonstration of their harmlessness and utility. A discussion followed on medical and surgical treatment in medico-legal cases, particularly in gunshot wounds of the brain, participated in by the following-named gentlemen, whose titles are also given : A 1, Dr. Hamilton D. Wey, Legal responsibility ; 2, Dr. E. G. Drake, Instruments nnd technique of operations ; 3, Dr. H. S. F. Ritter, Anesthetics and anesthesia ; 4, Dr. Charles L. Squire, The x-ray and other considerations ; 5, Dr. G. V. R. Merrill, The mortality and personal experience ; 6, Dr. Thomas A. Dundas, Post-mortem examinations ; B 7, Dr. G. M. Case, The relations of rheumatism and allied ailments to diseases of the eye and ear.

---

The Lake Erie Medical Society held its regular quarterly meeting at Dayton, Tuesday, January 25, 1898, under the presidency of Dr. N. E. Beardsley, of Dunkirk. The following program was observed: Election of officers. Papers: Hodgkin's disease, Dr. Joseph Rieger, Dunkirk; discussion by Drs. Thompson, Lattin and Ward. Dietetics, Dr. E. E. Davis, Forestville; discussion by Drs. B. S. Bourne, C. C. Johnson and French. A case of prostatitis, Dr. W. W. Jones, Dayton; discussion by Drs. Dodds, Lake and Bedient. Diagnosis and treatment of diphtheria, N. E. Beardsley, Dunkirk; discussion by Drs. Richmond, Rieger and Cherry.

---

The ninth international congress of hygiene and demography will be held at Madrid, April 10–17, 1898, under the patronage of his majesty, King Alfonso XIII. and her majesty the Queen Regent. The president of the congress is Dr. Julian Calleja and the secretary-general is Dr. Amalio Gimeno, minister of the interior, to whom all correspondence should be addressed.

---

The St. Louis Laryngological and Otological Society was organised December 27, 1897, composed of those physicians of St. Louis who limit their practice to the treatment of diseases of the nose, throat and ear. Dr. J. C. Mulhall was elected president, Dr. J. B. Shapleigh, vice-president, Dr. F. M. Rumbold, secretary, and Dr. A. S. Barnes, Jr., treasurer, for the year 1898. Meetings will be held monthly.

---

The American Medical Publishers' Association will hold its fifth annual meeting at Denver on Monday, June 6, 1898 (the day preceding the meeting of the American Medical Association). Editors and publishers, as well as everyone interested in medical journalism, are cordially invited to attend and participate in the deliberations. Several very excellent papers are already assured, but more are desired. In order to secure a place on the program, contributors should send titles of their papers at once to the secretary, Chas. Wood Fassett, St. Joseph, Mo.

